# Supplementary material for: Associations Between 40‐Year Trajectories of BMI and Proteomic and Epigenetic Aging Clocks: Deciphering Nonlinearity and Interactions
Source: Aging Cell. 2026 Jan 29;25(2):e70397. doi: 10.1111/acel.70397 (PMC12853144; doi:10.1111/acel.70397)

Table S1: Comparison of the EH-Epi subset with the rest of the Older Finnish Twin Cohort at recruitment, based on the 2011 questionnaire.

	In VpEpi data set	
	No	Yes
	N=8005	N=401
Sex		
Men	3586 (44.8%)	164 (40.9%)
Women	4419 (55.2%)	237 (59.1%)
Age at response 2011 (years)	60.3 (3.8)	60.2 (3.7)
When was your blood pressure last measured?		
less than half year ago	5364 (67.4%)	292 (72.8%)
half year – less than 1 year ago	1286 (16.1%)	74 (18.5%)
1 year – less than 5 years ago	1105 (13.9%)	30 (7.5%)
5 years or more than 5 years ago	193 (2.4%)	5 (1.2%)
never	16 (0.2%)	0 (0.0%)
Has a doctor ever told you that you have high blood pressure		
No	3933 (49.4%)	193 (48.1%)
Yes	4025 (50.6%)	208 (51.9%)
How often do you use blood pressure medications		
Never	4939 (65.2%)	241 (60.1%)
under 10 days last year	77 (1.0%)	0 (0.0%)
10 days to under 2 monhts	74 (1.0%)	0 (0.0%)
2-6 months	80 (1.1%)	2 (0.5%)

Over 6 months last year	2409 (31.8%)	158 (39.4%)
Body mass index in 2011 survey	26.2 (4.3)	26.3 (4.3)
Smoking status 2011		
never	3569 (45.7%)	201 (50.8%)
Occasional	491 (6.3%)	28 (7.1%)
Former	2332 (29.9%)	109 (27.5%)
Current	1417 (18.1%)	58 (14.6%)
Alcohol use as grams ethanol per day	15.6 (24.7)	16.2 (20.7)

Table S2: Description of the aging clocks used in the current study. **Caption:** All individuals with epigenetic ages also had proteomic ages. N: number of twins with available data.

Clock	Omic type	N	Original article	Description
PAC	Proteomic	401	Kuo et al., 2024a	This clock measures biological aging using Gompertz models with >100 blood proteins as predictors. Model training was conducted in a sample of the UK Biobank.
HPS	Proteomic	401	Kuo et al., 2024b	This clock estimates the 10-year risk of reaching the end of healthspan using Gompertz models with 86 blood proteins and age as predictors. Model training was performed on a sample from the UK Biobank. Unlike other clocks, HPS is negatively correlated with chronological age, as lower values indicate poorer prognosis.
ProtAge	Proteomic	401	Argentieri et al., 2024	The ProtAge Clock was designed to predict chronological age from blood proteins, 204 of which were used for its development in the UK Biobank. ProtAge showed strong predictive potential for major chronic diseases.
Adipose	Proteomic	401	Goeminne et al., 2025	The Adipose Clock is one of several organ-specific clocks built using blood proteins and elastic net regression in the UK Biobank to predict chronological age (1st generation). This clock is constructed to reflect the biological aging of adipose tissues.
Horvath	Epigenetic	379	Horvath, 2013	This pan-tissue clock estimates biological age based on DNA methylation patterns that best estimate chronological age across the genome. It is widely used to estimate biological age in various tissues, providing a general measure of aging.
Hannum	Epigenetic	379	Hannum et al., 2013	This clock uses DNA methylation to estimate chronological age in blood samples. It primarily estimates chronological age and correlates with mortality risk and age-related health outcomes.
PhenoAge	Epigenetic	379	Levine et al., 2018	This clock is based on a composite of clinical biomarkers of phenotypic age, which is predicted from DNA methylation patterns. It estimates mortality risk and captures an individual's physiological health and functional capacity.
GrimAge2	Epigenetic	379	Lu et al., 2022	This clock is built on nine DNAm-based surrogates of plasma proteins, DNAm PACKYRS, age and sex to estimate mortality risk. It predicts lifespan, mortality risk and the likelihood of developing age-related diseases.
DunedinPACE	Epigenetic	379	Belsky et al., 2022	This clock was trained on longitudinal physiological measurements of the pace of aging captured by DNA methylation at specific CpG sites. This clock predicts how fast an individual is aging relative to their chronological age, providing insight into future risk of age-related decline.

Characteristics of the biological clocks

The mean square error (MSE) for the ProtAge, Adipose, Horvath, Hannum, PhenoAge, and GrimAge2 clocks was 30.94, 41.18, 20.10, 54.59, 68.56, and 28.50, respectively. The Mean Absolute Error (MAE) for the ProtAge, Adipose, Horvath, Hannum, PhenoAge, and GrimAge2 clocks was 4.90, 5.33, 3.45, 5.99, 6.91, and 4.19, respectively. Epigenetic aging estimates, originally calculated in a larger sample of twins with a wide age range, correlated highly with chronological age in the Finnish Twin Cohort (ChronoAge) (Pearson r>0.90), except for DunedinPACE, which is a measure of the pace of aging and thus does not correlate with ChronoAge. Correlations with ChronoAge in the EH-Epi sample, for which the age range is lower, are presented below in addition to those of the proteomic clocks. Pairwise correlations between biological age clocks corrected for chronological age and scatter plots of biological age estimates versus chronological age are shown below as well.

Table S3: Pairwise correlations between nine biological aging clocks and chronological age (designated here as ChronoAge).

	PAC	HPS	ProtAge	Adipose	Horvath	Hannum	PhenoAge	GrimAge2	DunedinPACE	ChronoAge
PAC	1	-0.84	0.58	0.21	0.36	0.38	0.48	0.57	0.4	0.48
HPS	-0.84	1	-0.45	-0.3	-0.37	-0.36	-0.47	-0.64	-0.51	-0.38
ProtAge	0.58	-0.45	1	0.15	0.44	0.43	0.45	0.31	0.11	0.77
Adipose	0.21	-0.3	0.15	1	0.08	0.07	0.11	0.1	0.09	0.11
Horvath	0.36	-0.37	0.44	0.08	1	0.84	0.67	0.41	0.26	0.56
Hannum	0.38	-0.36	0.43	0.07	0.84	1	0.76	0.53	0.37	0.52
PhenoAge	0.48	-0.47	0.45	0.11	0.67	0.76	1	0.59	0.43	0.5
GrimAge2	0.57	-0.64	0.31	0.1	0.41	0.53	0.59	1	0.7	0.37
DunedinPACE	0.4	-0.51	0.11	0.09	0.26	0.37	0.43	0.7	1	0
ChronoAge	0.48	-0.38	0.77	0.11	0.56	0.52	0.5	0.37	0	1

Table S4: Pairwise correlations between biological aging clocks adjusted for chronological age.

	PAC	HPS	ProtAge	Adipose	Horvath	Hannum	PhenoAge	GrimAge2	DunedinPACE
PAC	1	-0.81	0.38	0.18	0.12	0.18	0.28	0.49	0.46
HPS	-0.81	1	-0.27	-0.28	-0.2	-0.21	-0.29	-0.58	-0.56
ProtAge	0.38	-0.27	1	0.1	0.03	0.07	0.11	0.04	0.17
Adipose	0.18	-0.28	0.1	1	0.03	0.01	0.07	0.07	0.09
Horvath	0.12	-0.2	0.03	0.03	1	0.78	0.49	0.26	0.31
Hannum	0.18	-0.21	0.07	0.01	0.78	1	0.8	0.43	0.42
PhenoAge	0.28	-0.29	0.11	0.07	0.49	0.8	1	0.54	0.56
GrimAge2	0.49	-0.58	0.04	0.07	0.26	0.43	0.54	1	0.75
DunedinPACE	0.46	-0.56	0.17	0.09	0.31	0.42	0.56	0.75	1

Table S5: Associations between waist circumference at wave 5 and aging clocks as assessed with generalized additive models. **Caption:** Aging clock estimates were adjusted for chronological age and residuals were scaled. ΔAIC is defined as the AIC of the nonlinear model minus the AIC of the linear counterpart, to indicate whether associations were nonlinear. %Dev: percentage of explained variation. R2: coefficient of determination. edf: effective degrees of freedom.

	**Aging clock**	**edf**	**F**	**p-value**	**%Dev**	**R2**	**ΔAIC**
Waist Circumference	PAC	2.0	5.7	1.66E-03	17.5%	16.2%	-0.14
Waist Circumference	HPS	2.6	31.3	<1.0E-16	21.3%	20.7%	-3.78
Waist Circumference	ProtAge	1.0	4.1	4.31E-02	1.0%	0.8%	0.00
Waist Circumference	Horvath	1.0	9.5	2.18E-03	2.5%	2.2%	0.01
Waist Circumference	Hannum	1.8	3.0	3.93E-02	2.2%	1.7%	0.35
Waist Circumference	DunedinPACE	2.5	17.5	<1.0E-16	13.4%	12.8%	-0.72
Waist Circumference	PhenoAge	2.3	6.8	2.38E-04	5.5%	4.9%	-0.71
Waist Circumference	Adipose	1.4	8.5	1.83E-03	3.5%	3.0%	0.49
Waist Circumference	GrimAge2	3.2	13.5	<1.0E-16	13.6%	12.8%	-5.06

Table S6: Associations between trajectories of BMI and aging clocks as assessed with generalized additive models when adding sex, smoking status and alcohol consumption as covariates. **Caption:** This table repeats the analyses presented in Table 2, but adds sex, smoking and alcohol consumption as covariates. Aging clock estimates were adjusted for chronological age and residuals were scaled. In models assessing the association between changes in BMI and biological aging, baseline BMI was added as a covariate, but model performance metrics (R^2, %Dev) exclude the effect of baseline BMI in the model. ΔAIC is defined as the AIC of the nonlinear model minus the AIC of the linear counterpart, to indicate whether associations were nonlinear. %Dev: percentage of explained variation. R2: coefficient of determination. edf: effective degrees of freedom. NS: Non-significant (p-value>0.05).

	Aging clock	edf	F statistic	p-value	%Dev	R2	ΔAIC
Baseline BMI	PAC	1.73	7.1	6.1E-04	17.6%	16.4%	0.53
Baseline BMI	HPS	2.22	8.9	1.8E-05	23.7%	22.4%	0.09
Baseline BMI	ProtAge	1.05	15.8	4.8E-05	7.4%	6.2%	0.10
Baseline BMI	Horvath	1.62	4.3	1.3E-02	6.2%	4.8%	0.48
Baseline BMI	Hannum	1.49	4.6	8.2E-03	8.6%	7.0%	0.48
Baseline BMI	DunedinPACE	1.83	6.8	6.2E-04	23.8%	22.5%	0.36
Baseline BMI	PhenoAge	1.00	9.5	2.2E-03	9.5%	8.2%	0.00
Baseline BMI	Adipose	1.00	8.1	4.7E-03	7.8%	6.4%	0.00
Baseline BMI	GrimAge2	1.00	4.7	3.0E-02	41.1%	40.2%	0.01
Change in BMI	PAC	3.50	2.1	6.6E-02	4.1%	4.1%	-3.51
Change in BMI	HPS	2.91	11.7	<1.0E-16	11.2%	11.2%	-0.74
Change in BMI	ProtAge	1.00	0.1	7.4E-01	0.1%	0.1%	*NS*
Change in BMI	Horvath	1.00	0.1	7.2E-01	0.1%	0.1%	*NS*
Change in BMI	Hannum	1.01	0.0	9.7E-01	0.4%	0.4%	*NS*
Change in BMI	DunedinPACE	1.00	26.0	9.1E-07	6.8%	6.8%	0.00
Change in BMI	PhenoAge	2.68	2.9	3.3E-02	3.7%	3.7%	-3.60
Change in BMI	Adipose	1.00	38.5	<1.0E-16	10.6%	10.6%	0.00
Change in BMI	GrimAge2	3.63	4.8	6.1E-04	6.6%	6.6%	-5.13
BMI (wave 5)	PAC	2.50	4.7	2.6E-03	17.6%	16.2%	-0.86
BMI (wave 5)	HPS	1.88	24.5	<1.0E-16	29.5%	28.4%	0.34

BMI (wave 5)	ProtAge	1.86	3.1	4.7E-02	5.1%	3.7%	0.51
BMI (wave 5)	Horvath	1.00	2.0	1.6E-01	4.4%	3.1%	*NS*
BMI (wave 5)	Hannum	1.12	0.9	2.9E-01	6.3%	4.8%	*NS*
BMI (wave 5)	DunedinPACE	1.00	41.8	<1.0E-16	28.5%	27.5%	0.00
BMI (wave 5)	PhenoAge	1.73	4.2	1.4E-02	9.8%	8.3%	0.23
BMI (wave 5)	Adipose	1.00	47.7	<1.0E-16	16.2%	15.0%	0.00
BMI (wave 5)	GrimAge2	2.17	5.2	1.8E-03	43.1%	42.0%	0.36

Figure S1: Study flowchart.

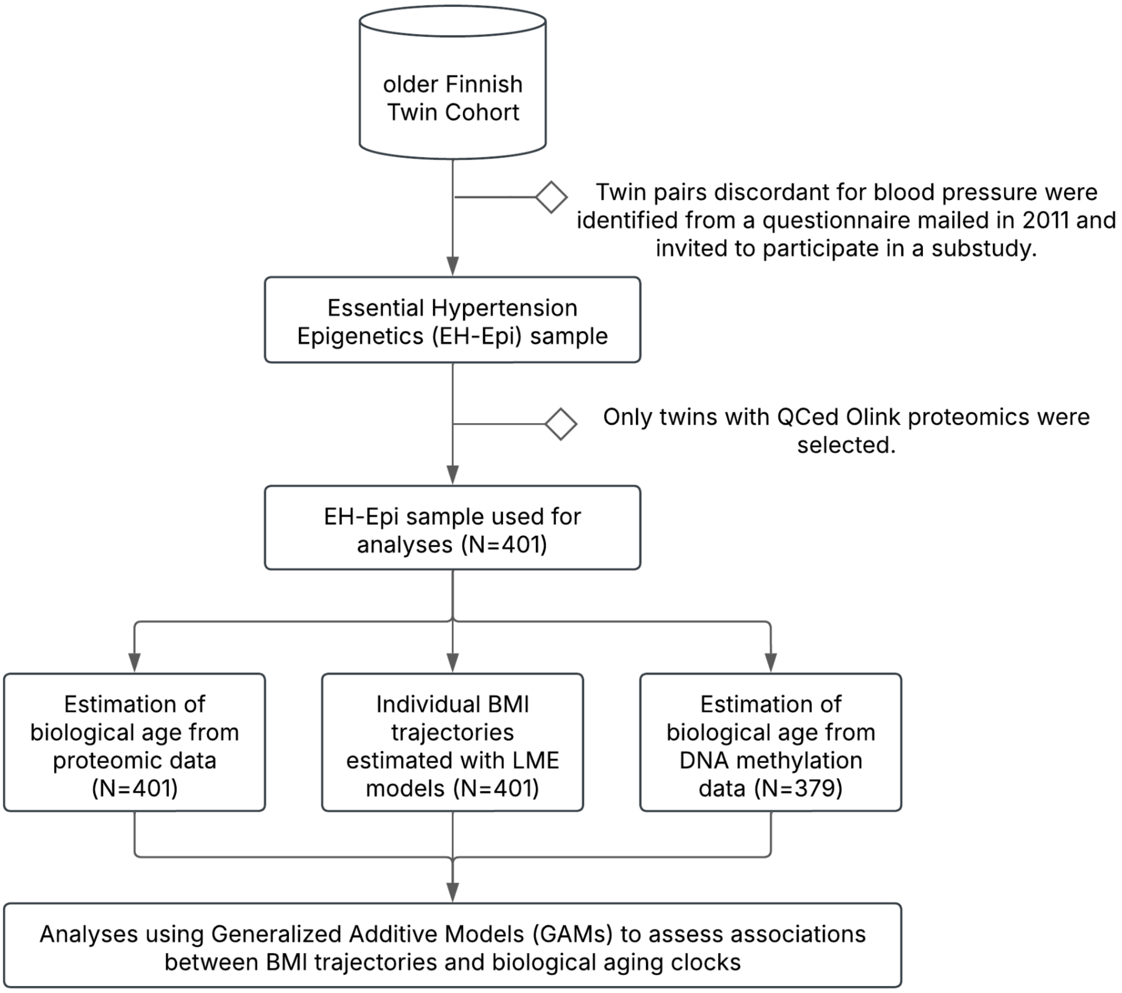

Figure S2: Scatter plots of biological age vs chronological age. DunedinPACE does not correlate strongly with chronological age because its values do not characterize estimates of biological age, but rather the pace of aging, a measure already correcting for chronological age.

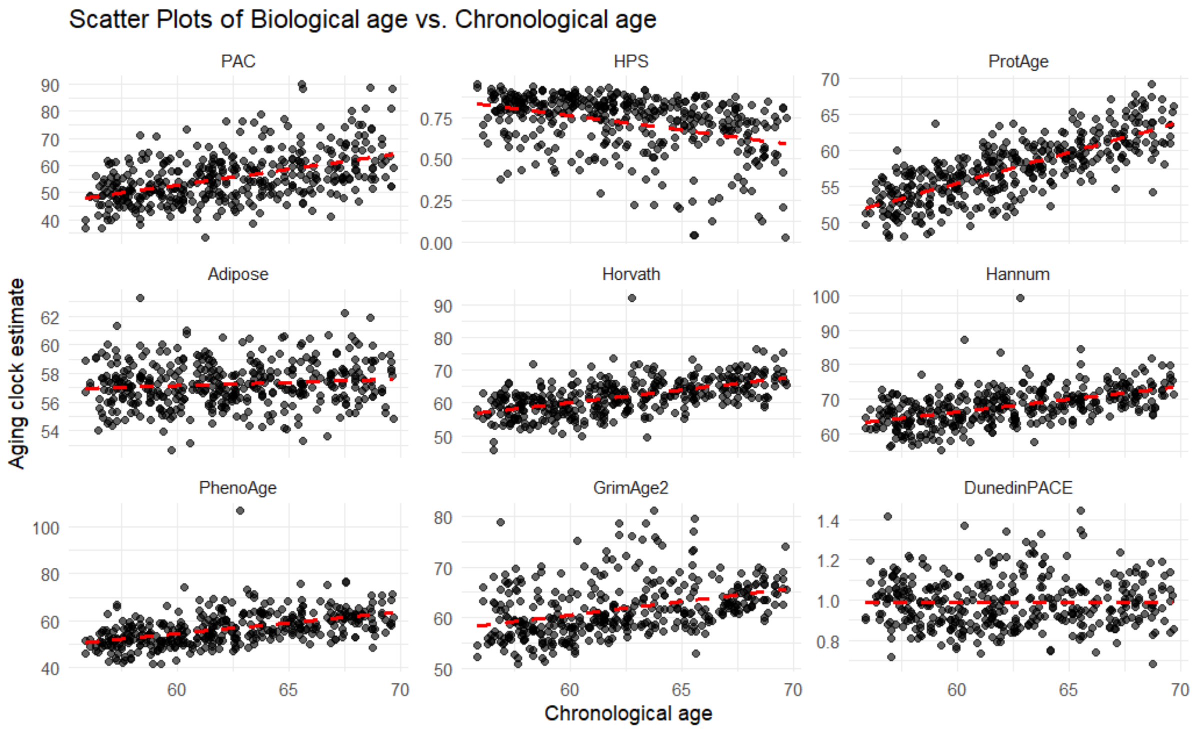

Figure S3: Individual body mass index trajectories over the follow-up period (on the left side) with the number of measurements per person given on the right-side panel

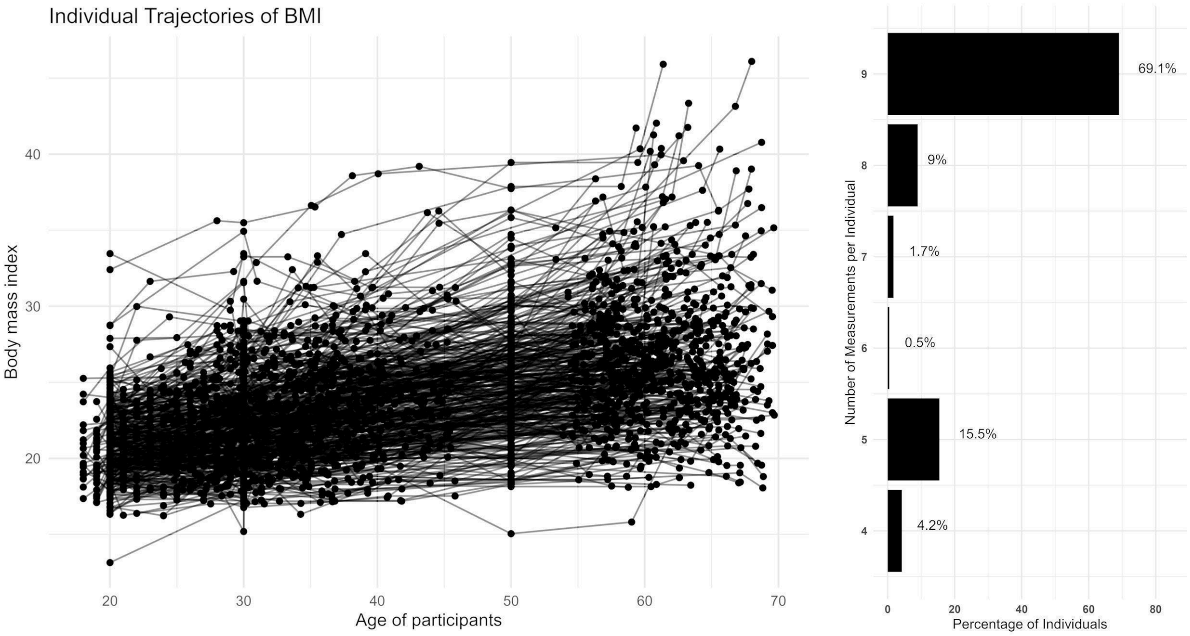

Figure S4: Graphical representation of associations between baseline BMI and biological aging from generalized additive models. **Caption:** Aging clock estimates were adjusted for chronological age and scaled. The y-axis represents the effect of baseline BMI on biological aging, which may vary across the range of baseline BMI values (x-axis). The significance of the associations and whether the models suggest that the associations are nonlinear are shown in Table 2.

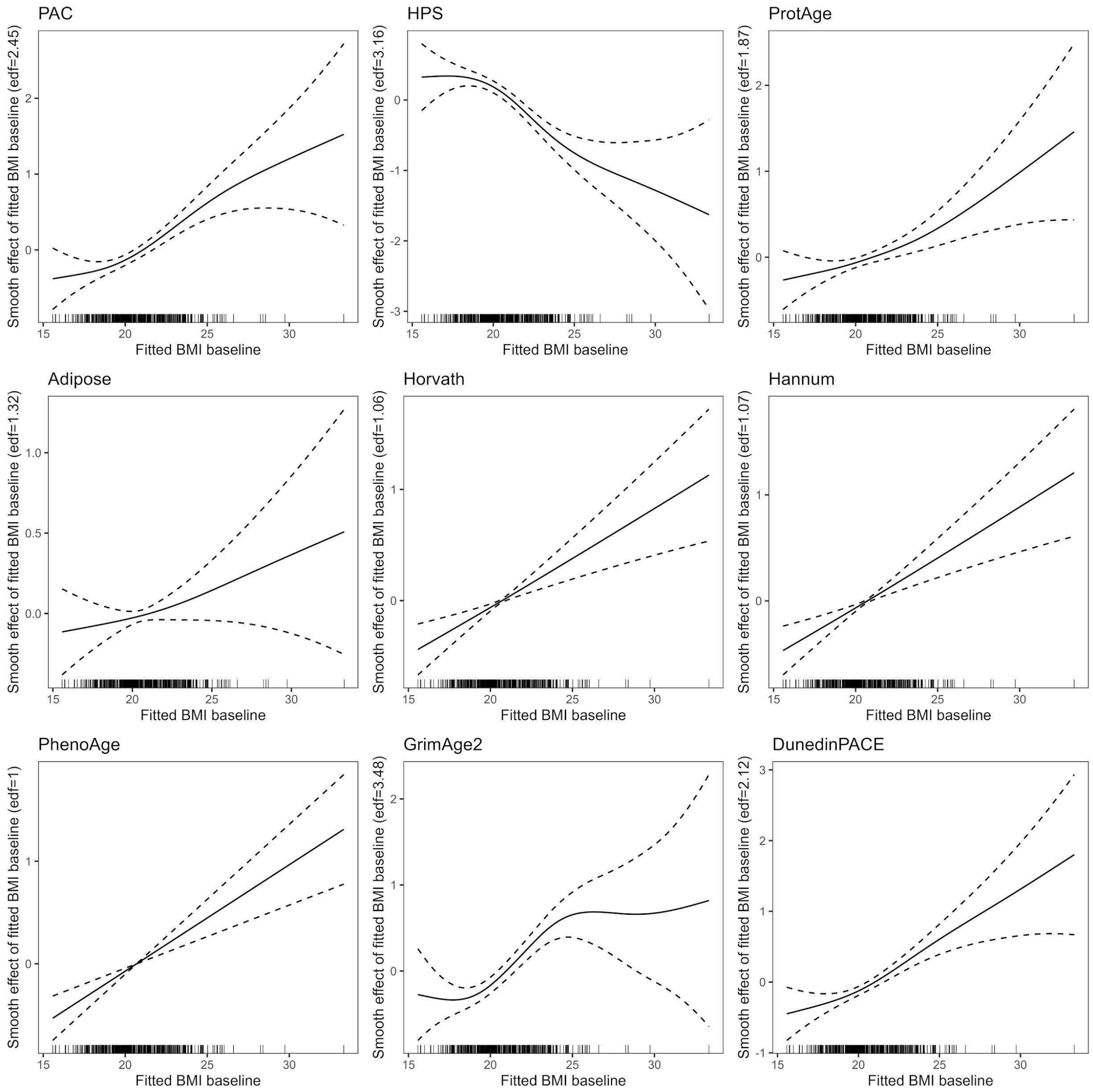

Supplement: Supplementary file 1 — Appendix S1: acel70397‐sup‐0001‐AppendixS1.pdf. [file ACEL-25-e70397-s001.pdf]
